# Pharmacological interventions for patients with chronic primary musculoskeletal pain: disparity between synthesized evidence and real-world clinical practice

**DOI:** 10.1097/PR9.0000000000001216

**Published:** 2024-12-09

**Authors:** Helen Koechlin, Cedric Werdelis, Antonia Barke, Beatrice Korwisi, Roland von Känel, Julia Wagner, Cosima Locher

**Affiliations:** a Department of Psychosomatics and Psychiatry, University Children's Hospital, University of Zurich, Zurich, Switzerland; b Division of Child and Adolescent Health Psychology, Department of Psychology, University of Zurich, Zurich, Switzerland; c Children's Research Centre, University Children's Hospital Zurich, University of Zurich, Zurich, Switzerland; d Department of Consultation-Liaison Psychiatry and Psychosomatic Medicine, University Hospital Zurich, University of Zurich, Zurich, Switzerland; e Division of Clinical Psychology and Psychological Intervention, Department of Psychology, University of Duisburg-Essen, Essen, Germany

**Keywords:** Electronic health records, Randomized controlled trials, Chronic primary musculoskeletal pain, Pharmacological treatment

## Abstract

**Introduction:**

Chronic primary musculoskeletal pain (CPMP) poses a major problem of public health, with high prevalence rates and economic burden. There is a wealth of clinical trials examining pharmacological interventions for patients with CPMP. Nevertheless, evidence from such trials does not necessarily mirror clinical realities.

**Objectives:**

We aimed to compare data sets from a clinical sample with an randomized controlled trial (RCT)-based sample.

**Methods:**

Both data sets included participants living with CPMP who received pharmacological interventions. The clinical sample was retrieved from electronic health records. The RCT-based sample stemmed from a network meta-analysis project. The following outcomes were used: demographic information, diagnosis-specific data, and pharmacological interventions (categorized according to the World Health Organization [WHO] analgesic ladder).

**Results:**

The clinical sample consisted of 103 patients (mean age: 50.25 years; SD: 14.0) and the RCT-based samples contributed 8665 participants (mean age: 51.97 years; SD: 6.74). In both samples, the proportion of women was higher than that of men (ie, 74.8% vs 58.9%). Psychiatric disorders were the most common comorbidities in the clinic sample but also the most frequent reason for patient exclusion in RCTs. The 2 samples differed significantly in medication classified as WHO III (clinical sample: 12.9%; RCT sample: 23.5%; *P* = 0.023) and WHO IV (clinical sample: 23.4%; RCT sample: 8.6%; *P* < 0.001), yet not WHO I and II.

**Conclusion:**

Our findings suggest a disparity between research-based study populations and clinical populations with CPMP. We advocate for future investigations on how to implement robust scientific evidence into real-world clinical practice, with a particular focus on addressing psychiatric comorbidities.

## Abstract 2

We shed light on both, data from real-life clinical practice and data from clinical trials to detect important disparities.

## 1. Introduction

The notion that chronic pain should always be understood as a biopsychosocial phenomenon is not a novel idea.^8,30,37^ The introduction of the International Classification of Diseases, 11th Revision (ICD-11) classification of chronic pain^106,107^ takes this concept into account, particularly with regard to the diagnostic entity of chronic *primary* pain. Chronic primary pain (CPP) is defined as pain that persists or recurs 3 or more months, is associated with significant emotional distress or functional disability, and that is not better accounted for by another chronic (secondary) pain diagnosis.^76^ For the category of chronic secondary pain, pain is considered a *symptom* of a health condition also classified in the ICD-11.^106^ A recent study estimated the prevalence rate of chronic pain to be as high as 20.4% among US adults, with 8% of those reporting high-impact pain (defined as pain that often limits life and work activities).^23^ Chronic primary musculoskeletal pain (CPMP), that is, pain in the tendons, muscles, bones, or joints, is a subgroup of CPP with especially high prevalence rates.^20^

In Switzerland, chronic (primary and secondary) musculoskeletal pain is one of the most frequent mentioned reasons for hospital visits and accounts for 13.4% of all treatments.^75^ Different treatment options exist to treat CPMP. Most common in clinical practice is the use of pharmacological interventions such as nonsteroidal anti-inflammatory drugs, muscle relaxants, antidepressants, opioids, anticonvulsants, and natural dietary supplements.^55,81^ However, the effect sizes of many pharmacological interventions are small to moderate compared with placebo.^1,14,19,31,91,95^ Furthermore, research processes in clinical trials do not necessarily reflect clinical practice. This evidence practice gap, or the delay in translating research results into clinical practice, has received increasing attention over the last years across different medical disciplines.^29,42,60,73^ The fact that scientific evidence usually takes several years to arrive in clinics seems to have different reasons: First, clinical trials may not reflect clinical practice because of their restricted inclusion criteria (eg, exclusion of common comorbidities).^98,113^ Second, physicians also rely on their personal experience when prescribing drugs,^98^ which can aggravate changes in prescription routines. In clinical trials, investigators may have a specific bias towards the investigated drug.^67^ Third, the availability of a drug on the market may also influence the prescription rates in clinical practice.^114^ As a result, it usually takes several years from research review and synthesis to the first policy statement.^44^

Therefore, comparing randomized controlled trials (RCTs) data with clinical realities in the population (eg, age, gender), diagnosis-specific data (eg, comorbidities), and intervention prescriptions is relevant for both future clinical trials and the professional practice.^65^ Given that there actually are significant gaps between RCTs and clinical practice, knowledge about those gaps might aid an in-depth discussion of dissemination challenges and help ensure that future RCTs better represent clinical realities.^2^

The goal of the present study was to compare data from electronic health records of patients from a Swiss University Hospital (ie, University Hospital Zurich) with RCT-based information from a large database that includes extracted data on the complete range of pharmacological interventions for those pain syndromes that would now be classified as CPMP. More specifically, we examined potential differences between those 2 samples regarding sample characteristics such as age and gender, use of medication groups, and comorbidities.

## 2. Methods

### 2.1. Clinical sample based on electronic health records

We used electronic health records data from patients of the University Hospital Zurich, Switzerland, admitted between November 2019 and November 2021. The Local Ethics Committee of Zurich, Switzerland, approved the design of the study (number: 2021-02101). We included all adult patients with a diagnosis of a pain syndrome that would be considered CPMP. To find the respective records, we used a 3-step approach: First, we focused on clinics within the University Hospital that potentially treat patients with CPMP (ie, anesthesiology, psychiatry and psychosomatics, rheumatology, complementary and integrative medicine, and neurology). Second, from these clinics, we included hospital discharge reports, rheumatology reports, case reports from chronic pain treatment, and initial psychosomatic initial interviews. Third, we further narrowed down the number of records by including only those that (1) mentioned an International Classification of Diseases, 10th Revision (ICD-10) diagnosis that would be classified as CPMP under the ICD-11 criteria; specifically F codes (mental and behavioral disorders), M codes (diseases of the musculoskeletal system and connective tissue), and R codes (symptoms, signs, and abnormal clinical and laboratory findings, not elsewhere classified), and (2) included at least 1 medication from the following classes: paracetamol/acetaminophen, nonsteroidal anti-inflammatory drugs (eg, phenylbutazone, ibuprofen), antiepileptics/anticonvulsants (eg, gabapentin, pregabalin), opioids (eg, morphine, oxycodone, naloxone), antidepressants (eg, selective and nonselective monoamine reuptake inhibitors, selective serotonin reuptake inhibitors, homeopathic and anthroposophical antidepressants, herbal medicines [ie, St. John's wort or combinations thereof]), antiphlogistic/antirheumatics in combination (eg, specific antirheumatics such as methotrexat, penicillamine), muscle relaxants (eg, tubocurarine, botulinum type A and B), antipyretics (eg, acetylsalicylic acid, kalium salicylate), or other medical preparations for musculoskeletal disorders (eg, vitamin B_12_, amid). Medications mentioned in the patient records were classified based on the World Health Organization (WHO) analgesic ladder^117^ into 1 of the 3 steps of the original ladder: nonopioid plus optional adjuvant analgesic for mild pain (step 1); weak opioid plus nonopioid and adjuvant analgesics for mild to moderate pain (step 2); and strong opioid plus nonopioid and adjuvant analgesic for moderate to severe pain (step 3). An optional fourth step includes (minimally) invasive interventions such as local anesthetic or nerve blocks.^3^ For our analyses, we focused on the WHO steps and not the individual medication classes.

In addition to the CPMP diagnosis and medication, we also extracted the following information from electronic health records: age, gender, comorbidities, and functional disability. We classified comorbidities into the following groups: cardiovascular (including everything from high blood pressure to congestive heart failure), pulmonary (from chronic cough to chronic obstructive pulmonary disease), dermatological (from folliculitis to systemic lupus erythematosus), hepatic (from steatohepatitis to cirrhosis of the liver), renal (from nephrolithiasis to chronic kidney disease), endocrinological (from hypothyroidism to diabetes), psychiatric (from mild depressive episode to major depressive disorder), chronic infection (from urinary tract infection to tuberculosis), neoplasia (from thyroid carcinoma to metastasized melanoma), and gastrointestinal (from chronic abdominal pain to Morbus Crohn).

One of the authors (C.W.) then scrutinized all identified electronic health records and classified them as “most likely CPMP,” “probably CPMP,” “unclear if CPMP,” “unlikely CPMP,” or “very unlikely CPMP.” A second author (H.K.) independently classified a randomly selected subsample of 20% of the cases. All disagreements were resolved by the study team. We calculated Cohen's Kappa to ascertain the interrater agreement, and interpreted >0.8 as almost perfect, >0.6 as substantial, >0.4 as moderate, >0.2 as modest, 0 to 0.2 as minor, and <0 as inadequate agreement.^58^

### 2.2. Randomized controlled trial–based sample

For the RCT-based sample information, we used data from a project that examines efficacy and safety of pharmacological interventions for CPMP by means of network meta-analysis. The project has been described in detail elsewhere.^54^ In brief, we included all RCTs identified through a systematic search (from inception until October 2022) that compared any pharmacological intervention with a second intervention or a control condition, and that aimed at patients with a pain syndrome that would now be classified as CPMP. In pairs of 2 researchers, we extracted information on study design, study characteristics, participant characteristics, intervention and control group details, outcome measures, and rated the risk of bias of preselected results using the Cochrane Risk of Bias Tool.^102^

### 2.3. Analysis

We compared the 2 samples (1 from electronic health records and 1 from RCT-based sample) with regard to age and gender distribution, frequency of use of the different medication groups (as classified with the WHO analgesic ladder), and comorbidities. In the RCT-based data, we used the list of exclusion criteria to account for comorbidities that would prevent patients from participating in a trial. The χ^2^ tests were calculated to detect differences between the samples, with *group* (ie, clinical or RCT-based sample) being the independent variable, and the variable of interest (ie, % female, WHO ladder I-IV) the dependent variable. Furthermore, we visually inspected differences between the samples by means of plots.

## 3. Results

### 3.1. Clinical sample based on electronic health records

**Figure 1.**
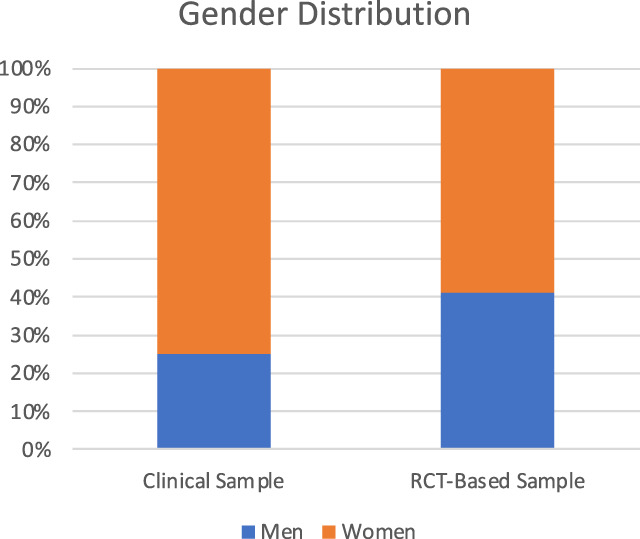
Gender distribution. RCT, randomized controlled trial.

**Figure 2.**
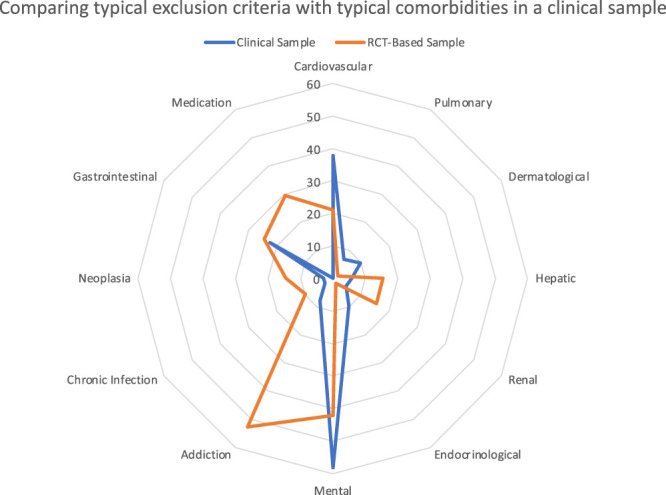
Comparing typical exclusion criteria with typical comorbidities in a clinical sample. RCT, randomized controlled trial.

**Figure 3.**
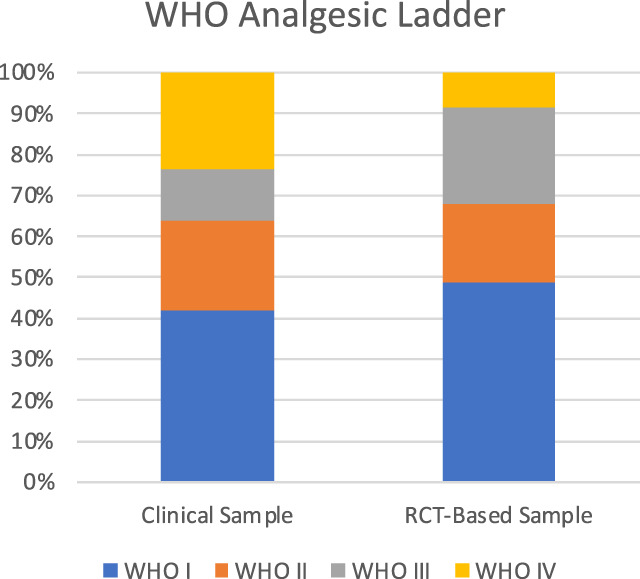
WHO analgesic ladder. RCT, randomized controlled trial; WHO, World Health Organization.

The clinical sample consisted of 103 patients: 77 women and 26 men with a mean age of 50.3 (SD: 14.0) years (Table 1 and Fig. 1). Cohen's Kappa between the 2 raters was 0.81 (95% confidence interval 0.802–0.828), that is, almost perfect.^58^ Functional disability, when reported, was used to judge whether a certain pain syndrome could be considered CPP or rather CSP. Comorbidities reported in electronic health records of patients were (in descending order of frequency) psychiatric disorders, cardiovascular disorders, vitamin D deficiency, gastrointestinal disorders, dermatological and endocrinological diseases, addiction, pulmonary diseases, hepatic diseases, renal diseases, chronic infections, and neoplasia (Fig. 2). The categories addiction and vitamin D deficiency were added during the classification process because of their noticeable frequency. On average, each patient had 2.19 comorbidities. In the electronic health record sample, many patients were treated with pain medication from the first step of the analgesic ladder (WHO I; 52 patients). In addition, 27 patients received WHO II medication, 16 patients were administered WHO III medication, and 29 patients were prescribed WHO IV medication (Table 1 and Fig. 3).

**Table 1:** Demographic information of the clinical sample and the randomized controlled trial sample.

	Clinical sample (N = 103)	RCT sample: 57 studies, 54 study arms* (N = 8665)	Difference χ^2^ test	
			χ^2^(*df*)	*P*
Gender, n (%)				
Female	77 (74.8)	5032 (58.9)	2.49 (1)	0.115
Male	26 (25.2)	3515 (41.1)		
Age (y), mean (SD)	50.25 (14.0)	51.97 (6.74)	NA	NA
Analgesic ladder				
WHO I	52 patients (41.9%)	19 study arms 3065 patients (48.7%)	0.81 (1)	0.369
WHO II	27 patients (21.8%)	5 study arms 1206 patients (19.2%)	0.35 (1)	0.552
WHO III	16 patients (12.9%)	5 study arms 1478 patients (23.5%)	5.17 (1)	0.023
WHO IV	29 patients (23.4%)	11 study arms 544 patients (8.6%)	24.05 (1)	<0.001

Significant at 95% confidence interval.

* Study arms without medication (ie, purely placebo arms) have been excluded from analyses.

NA, not applicable; RCT, randomized controlled trial; WHO, World Health Organization.

### 3.2. Randomized controlled trial–based sample

The patient population from the 57 studies^4,7,9,11,12,15–18,21,22,24,26,27,32,34,38,43,46–48,50,51,53,56,57,61–63,66,68–72,74,77,78,80,83–86,90,92,94,96,99–101,104,111,112,119,121–123^ [Note: O'Donnell et al., 2009 report 2 RCTs] of our RCT-based data consisted of 8665 participants, 5032 (58.9%) women and 3515 (41.4%) men, with a mean age of 51.97 (SD: 6.74) years (Table 1 and Fig. 1). With regard to comorbidities, studies excluded patients because of the following diseases (in descending order of frequency): psychiatric (26 studies), addiction (21 studies), cardiovascular (16 studies), neoplasia (13 studies), renal (7 studies), hepatic (7 studies), gastrointestinal (5 studies), pulmonary (4 studies), chronic infection (3 studies), endocrinological (2 studies), and dermatological (1 study). None of the studies excluded patients based on vitamin D deficiency. In addition, 13 studies excluded all patients who were currently on medications for psychiatric disorders, and 16 studies excluded patients with uncontrolled disease activity (eg, uncontrolled mental or somatic disorders or any condition that could interfere with the interpretation of the outcome assessment) (Fig. 2). With regard to the WHO analgesic ladder, 19 study arms examined medications categorized as WHO I (N = 3065), 5 study arms as WHO II (N = 1206), 5 study arms as WHO III (N = 1478), and 11 study arms as WHO IV (N = 544). Finally, 14 study arms examined other medication (N = 1046; Table 1 and Fig. 3).

### 3.3. Sample comparison

The comparison of the clinical sample and the RCT-based sample revealed that the 2 populations did not differ in their gender distribution, with both samples having a greater proportion of women when compared with men (clinical sample: 74.8% vs RCT-based sample: 58.9%; χ^2^(1) = 2.49, *P* = 0.115). In the intake of medications, our samples did not differ in medication classified as WHO I (clinical sample: 41.9%; RCT sample: 48.7%; χ^2^(1) = 0.81, *P* = 0.369) and WHO II (clinical sample: 21.8%; RCT sample: 19.2%; χ^2^(1) = 0.35, *P* = 0.552). However, the 2 samples differed significantly in medication classified as WHO III (clinical sample: 12.9%; RCT sample: 23.5%; χ^2^(1) = 5.17, *P* = 0.023) and WHO IV (clinical sample: 23.4%; RCT sample: 8.6%; χ^2^(1) = 24.05, *P* < 0.001). See Table 1 and Figures 1–3 for details.

## 4. Discussion

The aim of this study was to compare data from electronic health records with data extracted from RCTs and to determine differences between these samples with regard to several characteristics in adult patients with CPMP. We found notable similarities in the samples regarding gender distributions. In both clinical trials and RCTs, the proportion of women was greater—consistent with the observation that chronic pain is more common in women than in men across all age groups.^10,20,52,59^ Several contributors to this sex and gender differences have been shown to influence the pain experience,^79^ ranging from differences related to hormones and genetics to psychosocial factors such as socialization related to gender norms and societal expectations of gender role expectations (for a review see Samulowitz et al.^89^). Furthermore, it is crucial to note that social disparities significantly contribute to differences in pain prevalence and disproportionately disadvantage groups based on gender, education, and wealth.^41^ We were unable to examine these differences in our data because neither electronic health records nor RCT-based data reliably included sociodemographic background information. Although most of the RCTs report participants' age and gender (in line with guidelines, eg, CONSORT^93^), variables such as ethnicity, level of education, socioeconomic status, or occupation are presented much less frequently.^97,105^ This has resulted in insufficient representation of diverse populations, leading to calls for heightened inclusion of historically marginalized communities in research.^110,116^

Importantly, patient characteristics that (potentially) affect the risk or benefit of a treatment may significantly affect the generalizability of trials results, if these characteristics differ between the selected study population and the patient group to which the results are applied.^124^ Specifically, comorbidities may have a severe impact on risk and benefit profiles of treatments. In fact, this is the main argument to exclude participants with these comorbidities from RCTs in a bid to maximize internal validity and to minimize risk for the participating patients. It is certainly no coincidence that the list of exclusion criteria mirrors the list of the comorbidities observed in clinical practice. In clinical practice and noninterventional studies, there is high occurrence of comorbidity between chronic pain and psychiatric disorders, particularly with anxiety disorders and depression.^5,103,109^ In clinical trials, however, eligibility criteria are often narrow and exclude patients with comorbidities—a well-known phenomenon throughout the medical field (for a review, see Van Spall et al.^113^), although the proportion of patients with 2 or more medical conditions is increasing.^115^ Furthermore, research shows that the associations between chronic pain syndromes and psychiatric comorbidities are likely bidirectional.^25,40,49^ The concomitant occurrence of chronic pain and psychiatric disorders might even influence the effectiveness of antidepressants.^88^ Likewise, mental health conditions can significantly influence pain perception and treatment response, and it has been argued that the efficacy of pain medications observed in RCTs may not fully translate to real-world clinical settings.^39^ Hence, effective pain treatment should adopt a biopsychosocial framework that also addresses the psychiatric needs of patients with chronic pain. It is therefore questionable to what extent the results of RCTs can be generalized to the clinical population of interest.

As is established, intervention research faces the well-known tension between maximizing internal or external validity. This tension is reflected in the distinction between efficacy and effectiveness trials. Although efficacy trials determine the effect of an intervention under *ideal* circumstances, effectiveness trials measure the effect under *real-world clinical* settings.^35^ These 2 types of trials differ with regard to their primary goal, the population they include, the outcomes they are interested in, study duration, and the assessment of adverse events.^36^ An RCT provides a good framework for efficacy evaluation because bias is minimized through mechanisms such as blinding and randomization.^98^ The downside, however, is that the external validity of the results gained through RCTs is less than perfect, which means that the extent of applicability to clinical practice varies widely^87^; this is also reflected in our results regarding psychiatric comorbidities. Therefore, there is a growing inclination to assess intervention effectiveness within the context of *real-life circumstances* in routine clinical practice, for example, through so-called pragmatic trials.^82^ Pragmatic trials are characterized by being embedded into ongoing clinical practice, flexible treatment protocols, and outcomes that reflect clinical interest (eg, disability, risk-benefit analysis). Thus, they aim to directly inform health care decision making.^13,33,45,108^ Pragmatic trials are especially promising as complements to RCTs, for example following a phase III drug trial. Despite the advantages of pragmatic trials, a recent systematic review of pragmatic trials of pain treatments identified areas in which current practices in such trials could be improved, for example, regarding patient recruitment.^45^

We have previously argued that the assessment of emotional distress is key in chronic pain trials.^6^ This is also in line with guidelines for outcome reporting in chronic pain trials such as the IMMPACT recommendations,^28^ clearly advising the assessment of emotional functioning. Furthermore, tools that help design RCTs, such as the PRECIS-2 tool,^64^ also point out that the choice of outcome should be guided by the question; that is, to what extent is the trial's primary outcome directly relevant to participants? Given that emotional distress is a primary diagnostic criteria for CPP,^76,106^ RCTs can no longer neglect this important domain. The results of our present study, pointing out the gap regarding comorbidities between clinical trials and clinical practice, provide additional support for the proposition that RCTs should increase efforts to incorporate assessments of emotional distress.

Apart from demographic variables, we found a noteworthy trend on the types of medications under examination in RCTs. The majority of studies focused on medications classified as WHO I, with WHO IV being the second most frequently reported category. In contrast, studies involving medications falling under WHO II and III were less common with only 5 study arms per classification. Notably, there were important differences between our samples in medication. Whereby WHO III medications were significantly less present in the clinical sample, WHO IV medications were significantly more common when compared with the RCT-based sample. For example, a large difference occurred for the WHO IV medication (ie, [minimally] invasive interventions such as local anesthetic or nerve blocks). A total of 23.4% of patients reported to take such medication, whereby only 8.6% of participants from clinical trials were under investigation for this drug. This discrepancy is in line with the finding that clinical practice for the prescription of invasive interventions is in contrast to guidelines; clinical guidelines clearly recommend interdisciplinary pain treatment that include both pharmacological and nonpharmacological approaches.^118^

### 4.1. Limitations

This study has several limitations. First, because of data availability, our study compared data from a clinical sample that is solely treated in a single University Hospital in Switzerland, whereas the data from the RCT-based sample came from various countries. Second, we did not examine the influence of factors on medication adherence.^120^ Third, and related to our study design, the sample sizes of our 2 data sets (ie, electronic health records data vs RCT-based data) are very different, resulting in imbalanced groups. Fourth, with regard to psychiatric comorbidities, it remains unclear how consistently and reliably especially mild diagnoses have been reported in the electronic health records. Finally, we had to rate all ICD-10 diagnoses in their likelihood to be classified as CPMP according to the novel ICD-11 criteria (ie, ranging from “very unlikely CPMP” to “most likely CPMP”). Thus, in all cases, there remains a certain level of uncertainty regarding the fulfillment of the ICD-11 criteria. Notably, our Kappa statistic demonstrated a nearly perfect level of interrater agreement.

### 4.2. Conclusions

Our findings suggest that individuals with CPMP who are enrolled in clinical trials do not comprehensively mirror patients from real-world clinical settings in comorbid conditions and medication prescription practices. Thus, in the future, we hope for more studies that foster the external validity by including a diverse sample of patients and reflecting clinically relevant comorbidities. This may help to improve the transferability of the results into clinical practice.

## Disclosures

B. Korwisi received consulting fees from International Association for the Study of Pain (IASP), outside the submitted work. The remaining authors have no conflicts of interest to declare.
